# Local and Global Changes in Brain Metabolism during Deep Brain Stimulation for Obsessive-Compulsive Disorder

**DOI:** 10.3390/brainsci9090220

**Published:** 2019-08-30

**Authors:** Juan Carlos Baldermann, Karl Peter Bohn, Jochen Hammes, Canan Beate Schüller, Veerle Visser-Vandewalle, Alexander Drzezga, Jens Kuhn

**Affiliations:** 1 Department of Psychiatry and Psychotherapy, University of Cologne, Medical faculty, 50937 Cologne, Germany; 2 Department of Nuclear Medicine, University of Cologne, Medical faculty, 50937 Cologne, Germany; 3 Department of Stereotactic and Functional Neurosurgery, University of Cologne, 50937 Cologne, Germany; 4 Department of Psychiatry, Psychotherapy and Psychosomatic, Johanniter Hospital Oberhausen, 50937 Oberhausen, Germany

**Keywords:** Deep brain stimulation, DBS, Obsessive-Compulsive Disorder, OCD, Positron emission tomography, 18F-fluorodeoxyglucose, FDG-PET

## Abstract

Recent approaches have suggested that deep brain stimulation (DBS) for obsessive-compulsive disorder relies on distributed networks rather than local brain modulation. However, there is insufficient data on how DBS affects brain metabolism both locally and globally. We enrolled three patients with treatment-refractory obsessive-compulsive disorder with ongoing DBS of the bilateral ventral capsule/ventral striatum. Patients underwent resting-state ^18^F-fluorodeoxyglucose and positron emission tomography in both stimulation ON and OFF conditions. All subjects showed relative hypometabolism in prefronto-basal ganglia-thalamic networks compared to a healthy control cohort when stimulation was switched OFF. Switching the stimulation ON resulted in differential changes in brain metabolism. Locally, volumes of activated tissue at stimulation sites (*n* = 6) showed a significant increase in metabolism during DBS ON compared to DBS OFF (Mean difference 4.5% ± SD 2.8; *p* = 0.012). Globally, differential changes were observed across patients encompassing prefrontal increase in metabolism in ON vs. OFF condition. Bearing in mind limitations of the small sample size, we conclude that DBS of the ventral capsule/ventral striatum for obsessive-compulsive disorder increases brain metabolism locally. Across distributed global networks, DBS appears to exert differential effects, possibly depending on localization of stimulation sites and response to the intervention.

## 1. Introduction

Obsessive-compulsive disorder (OCD) is among the most common psychiatric disorders with a lifetime prevalence of 2–3 % [1]. Characterized by the presence of unwanted and aversive thoughts (obsessions) and consequent repetitive neutralizing actions (compulsions), it is a chronic, often severely disabling disorder.

For severely affected patients that do not respond to first-line treatments such as cognitive behavioral therapy and pharmacotherapy, deep brain stimulation (DBS) for OCD has been investigated for over two decades. Despite clinical utility of the intervention and approval by the U.S. Food and Drug Administration (FDA) [2], the underlying mechanisms of the intervention are scarcely known.

Beyond an ongoing debate on which target may be best to treat OCD, recent approaches have suggested that clinical effects may be driven by broader network changes rather than focal brain modulation [3]. This notion is supported by reports of network modulations in DBS for other disorders such as Parkinson’s disease (PD) or Alzheimer’s disease [4,5]. However, there is insufficient data on how DBS for OCD affects brain functioning and metabolism both locally as well as in in broader global networks.

Functionally, Figee et al. have reported that DBS of the VC/VS decreased excessive connectivity between the ventral striatum and the prefrontal cortex [6] and that decrease in connectivity is associated with overall treatment response in the Yale-Brown Obsessive-Compulsive Scale (Y-BOCS). A study using oxygen-15 positron emission tomography (^15^O-PET) showed that DBS ON compared to switched DBS OFF resulted in an increase of perfusion in the dorsal anterior cingulate cortex (dACC) and the basal ganglia, depending on the employed stimulation contacts of the DBS electrodes [7]. Similar to this, an early case report using ^15^O-PET described an increase in activity in the dorsolateral prefrontal cortex (DLPFC) and the cingulate cortex [8]. Contradicting these observations, Suetens et al. observed a decrease in brain metabolism using ^18^F-fluorodeoxyglucose and positron emission tomography (FDG-PET) in the ACC, the medial frontal gyrus, and in the right temporal gyrus in DBS ON vs. DBS OFF condition [9]. 

Overall, there is some evidence that DBS of the VC/VS induces changes in brain functioning beyond local and focal modulation around DBS electrodes. However, the data is limited and conflicting. Moreover, there is scarce data on the local effects of DBS OFF compared to DBS ON for OCD in humans.

Thus, we employed FDG-PET in a case series of patients with treatment-refractory OCD to further test how brain metabolism in both locally modulated brain areas and in broader connected areas respond to VC/VS-DBS. Specifically, we sought to investigate whole brain changes in metabolism as well as changes in the modelled electric fields based on individually applied stimulation programming.

## 2. Materials and Methods

### 2.1. Subjects

Derived from a larger clinical trial on VC/VS-DBS for OCD including 20 subjects ^10^, we offered patients to participate in the present FDG-PET study. Overall, five patients agreed to participate. Of these five patients, only three completed both ON and OFF conditions. In total, we enrolled three patients (two males, one female) with treatment-refractory OCD (see Table 1). The main reasons to reject study participation were concerns regarding radiation exposure and unwillingness to switch off the stimulation (due to anticipated symptom relapse). All included patients consented to the procedure according to the Declaration of Helsinki. The Ethics committee of the Medical Faculty of the University of Cologne and the radiation protection authorities approved the study (DRKS number: 00008583).

### 2.2. Surgical Procedure

**Figure 1:**
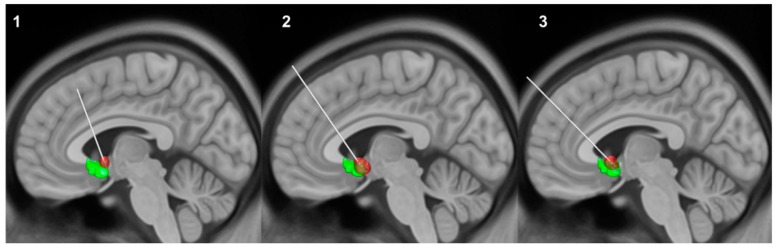
Overview of electrode localization of each individual subject (1–3) and corresponding volumes of activated tissue (VAT) (red) depending on stimulation settings at time of imaging acquisition. More distal contacts were implanted in the ventral striatum (green); more proximal contracts were located in the ventral capsule. Only left electrodes are shown for display purposes. For a closer view see  Figure S1.

Bilateral quadripolar electrodes (Model 3387 DBS Lead; Medtronic; Minneapolis, MN, USA) were stereotactically implanted bilaterally under local anesthesia. The two distal contacts (0, 1 on the left and 8, 9 on the right electrode, respectively) were placed in the nucleus accumbens within the ventral striatum bilaterally (see Figure 1 for an overview). The more proximal contacts (2, 3 and 10, 11) were located in the ventral part of the VC. Post-operative computer tomography (CT) was used to confirm the correct position of the electrodes post-operatively. For further description of the surgical procedure and patient inclusion criteria see [10].

### 2.3. PET Imaging and Analysis

Imaging acquisition was performed six to twelve months after DBS implantation with stable stimulation settings for at least four weeks. Each subjects underwent two scanning sessions: one with stimulation switched ON, one with stimulation switched OFF. The OFF condition measurement took place after the stimulation was switched off for 24 to 48 h. On average, four days passed between the two scanning sessions. In this period, medication remained stable (see Table 1) and no specific psychotherapy was applied.

FDG-PET scans were performed at the Department of Nuclear Medicine, University Hospital Cologne, Germany, with a Siemens Biograph mCT Flow 128 Edge scanner (Siemens, Knoxville, TN, USA). After the injection of 200 MBq [18F]-FDG, patients waited for 30 min lying in supine position in a room with dimmed light. After that, a static image was acquired over 15 min. A low-dose CT scan for the purpose of attenuation correction was acquired prior to PET acquisition. Scans were iteratively reconstructed using a 3-D OSEM algorithm (four iterations, 12 subsets, Gaussian filter: 5 mm full width at half maximum (FWHM), 400 × 400 matrix, slice thickness of 3 mm). The FDG scans were then spatially normalized to MNI space [11] to the FDG-PET template published by Della Rosa [12] with SPM 12 (www.fil.ion.ucl.ac.uk/spm/software/spm12). The resulting image resolution was 2 × 2 × 2 mm^3^ with matrix dimensions of 79 × 95 × 78 voxels. Standardized uptake value ratio (SUVR) image-datasets were created using the AAL-atlas cerebellum as reference region [13]. The cerebellum was chosen because changes in metabolism were expected in frontal cortical areas and the basal ganglia based on prior literature [6,7,9]. 

First, we assessed FDG-metabolism in DBS OFF condition for each subject by performing a voxel-wise comparison of individual subjects with an age-matched cohort of healthy subjects that is implemented in the NEUROSTAT software [14]. This publicly available healthy control group consisted of 22 subjects (7 men, 15 women). Thus, this control group does not constitute a separately recruited sample but rather a normative reference group. Second, intraindividual DBS induced changes in FDG-metabolism between DBS ON and OFF condition were calculated by voxel-wise subtraction. The resulting difference maps were displayed in VINCI 4 [15]. Third, mean FDG-metabolism in both conditions was extracted from volumes of interest (see Section 2.4) using a custom-built Matlab script (The MathWorks, Inc., Natick, MA, USA) for statistical analysis.

### 2.4. Reconstruction of Volume of Tissue Activated

Volumes of interests (VOI) were selected to test how DBS affects brain metabolism locally within the electric field induced by the DBS system. To build such models of individual volumes of activated tissue (VAT), we reconstructed patients’ electrodes and electric fields in standard space using LEAD-DBS (https://www.lead-dbs.org/) [16]. Briefly, postoperative CT scans were linearly coregistered to preoperative magnetic resonance imaging (MRI) using SPM 12. Coregistrations were manually controlled for each patient and refined if needed using Advanced Normalization Tools (http://stnava.github.io/ANTs/). Images were then normalized into ICBM 2009b NLIN asymmetric space using the SyN approach implemented in Advanced Normalization Tools. A subcortical brain shift correction was applied using LEAD-DBS if needed to attain a more precise subcortical alignment. DBS electrodes were localized within MNI space by the artifact in the postoperative image using Lead-DBS software.

Volumes of tissue activated (VTA) estimation protocol followed the one by Horn and colleagues [17]. In brief, individual stimulation parameters were used to model an electric field in dependence of the surrounding brain tissue following the FieldTrip-SimBio pipeline (http://fieldtriptoolbox.org). This created bilateral VTAs that were used as VOI in the quantitative PET analysis. We used SPSS (IBM Corp. Version 25.0. Armonk, NY, USA) for non-parametric Wilcoxon signed-rank testing of dependent samples to assess differences in glucose uptake of VOIs during DBS ON and OFF conditions.

## 3. Results

**Figure 2:**
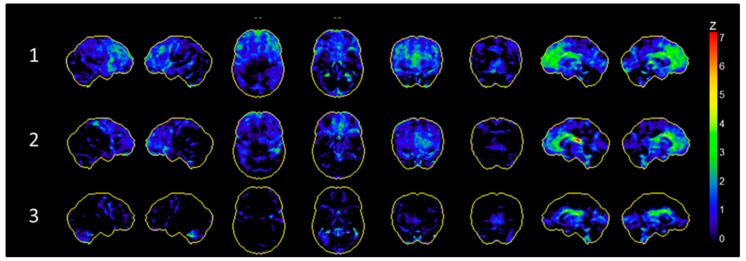
Glucose hypometabolism during stimulation OFF condition compared to an age-matched healthy control cohort. Patients showed most pronounced relative hypometabolism in the medial prefrontal cortex as well as the thalamus.

All three patients underwent FDG-PET imaging in both DBS ON and OFF condition. When DBS was switched OFF), all three subjects showed a relative frontal hypometabolism compared to a cohort of healthy control subjects. This hypometabolism was most pronounced in subject 1 and 2 in the medial prefrontal cortex as well as in the basal ganglia and thalamus (see Figure 2). There was no distinct global hypermetabolism compared to healthy controls across subjects (see Figure S2 Supplementary Materials).

**Figure 3:**
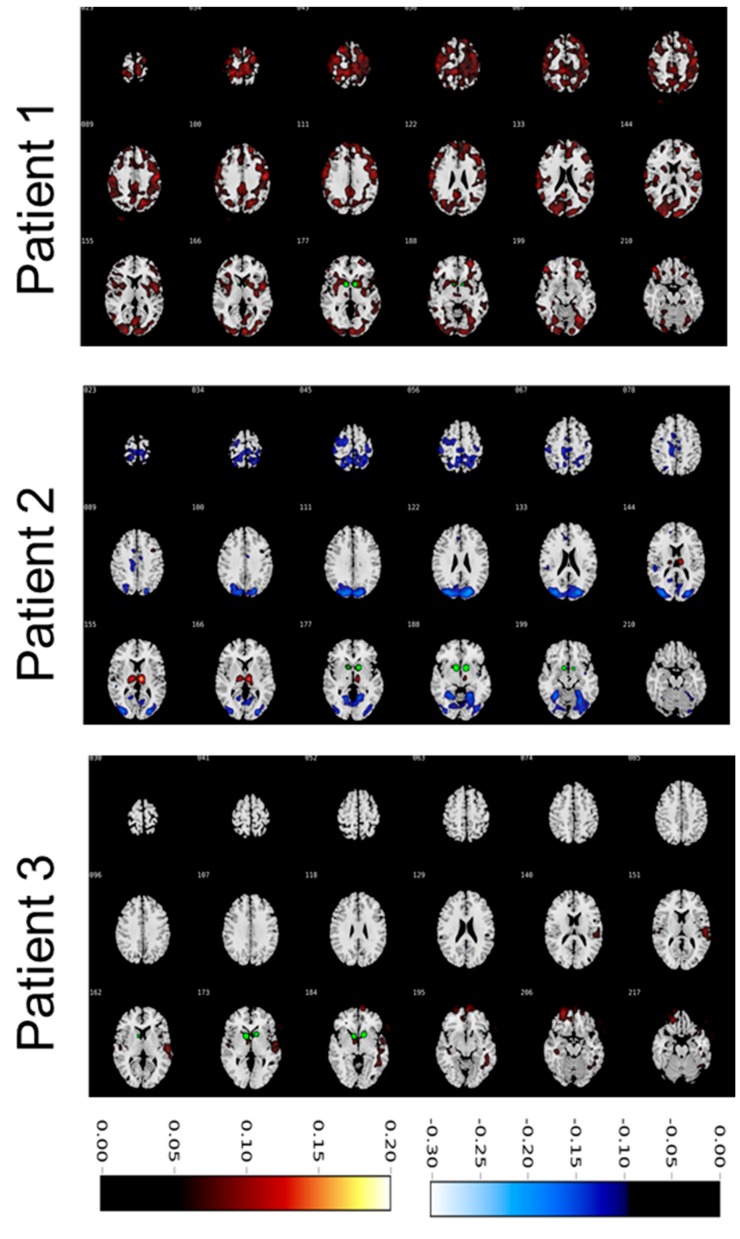
Ratios of glucose metabolism in deep brain stimulation ON vs. OFF condition. Warm colours indicate increased uptake in ON condition compared to OFF condition. Cold colours indicate increased metabolism in OFF condition compared to ON condition. Colour bars represent ratios in standardized uptake values. Volumes of activated tissue are displayed in green.

To assess changes in glucose metabolism induced by ongoing VC/VS-DBS, we calculated differential contrasts between DBS ON and OFF conditions. Global changes differed substantially between subjects (see Figure 3). Subject 1 revealed an increase in glucose uptake in ON compared to OFF condition in the frontal, parietal, and occipital lobes. Increased uptake was also present in the ventral striatum and caudate nucleus. Contrary to this, subject 2 showed a marked hypometabolism in ON compared to OFF condition in the frontal and parietal cortex. Additionally, we observed a pronounced increase in glucose uptake in the thalamus. Of note, the thalamus of subject 2 showed a strong hypometabolism in the OFF condition compared to healthy controls, thus this hypometabolism was diminished when switching the stimulation ON. Subject 3 showed a relative hypermetabolism in the orbitofrontal cortex in ON vs. OFF condition as well as in the temporal cortex. However, overall, differences between conditions were less overt in subject 3.

**Figure 4:**
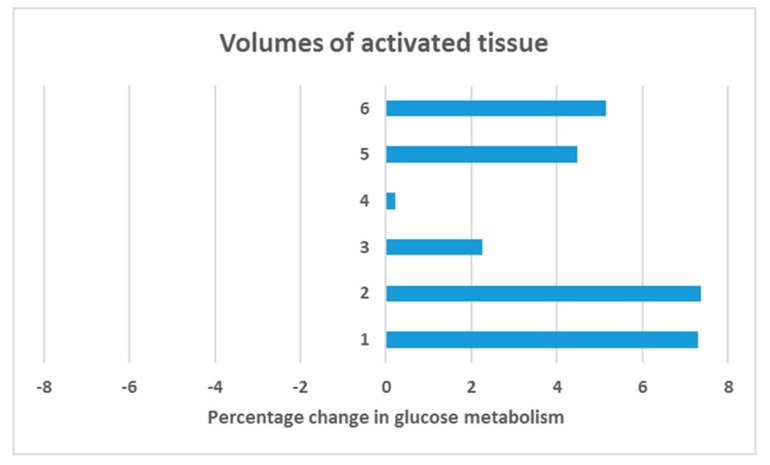
Volumes of interest analysis. We modelled volumes of activated tissue (VTA) based on the individually applied electric field per electrode, resulting in 6 VTAs for three subjects (VTA 1–2 = subject 1; VTA 3–4 = subject 2; VTA 5–6 = subject 3). Overall, a significant increase in glucose metabolism in VTAs of 4.4 % was observed when switching DBS ON compared to DBS OFF in a non-parametric Wilcoxon signed-rank test (Z = 2.201; SD = 2.6; *p* = 0.028).

In a VOI analysis we specifically assessed changes in glucose metabolism in the electric fields induced by the electrodes. Interestingly, we observed an increase of glucose uptake in all subjects and VTAs (see Figure 4) with a statistically significant increase of 4.4 % (Z = 2.201; SD = 2.6; *p* = 0.028).

## 4. Discussion

The aim of this add-on study [10] was to study how VC/VS-DBS influences brain metabolism in patients with treatment-refractory OCD. Specifically, we explored changes in brain stimulation in the individually, directly altered brain areas through the electric field induced by the electrodes. Furthermore, we wanted to display global changes beyond stimulation sites across the whole brain. Whereas some larger studies addressed this important topic in patients with PD (e.g., [18,19]), the existing literature in the field of DBS for OCD is scarce and conflicting [7,9]. DBS for PD is clinically more established than DBS for OCD which is only performed in a small subset of severely affected, treatment-resistant patients, which limits potential study participants. Another possible explanation could be that, as in our study, patients refuse to participate due to concerns regarding radiation exposure and unwillingness to switch off the stimulation and possibly experience a symptom relapse. Nonetheless we managed to obtain three complete datasets with two scans each, revealing remarkable stimulation effects.

To assess local changes in metabolism, we calculated individual electric fields determined by the placement of DBS leads and the individually employed stimulation settings. The resulting models form an estimation of the actual anatomical expansion of electric fields, taking into account the tissue around the electrodes and the corresponding current conduction (albeit not other potentially influencing factors such as local fibre orientation). Notably, all subjects displayed an increase in FDG metabolism in each VAT (see Figure 4). Across all subjects, we observed a significant mean increase in FDG uptake of 4.4 (± 2.6) percent. We can therefore conclude that VC/VS-DBS increased glucose brain metabolism locally. Interestingly, similar effects of local stimulation-induced brain hypermetabolism have been observed in DBS of the nucleus basalis of Meynert [20]. Of note, these patients received low-frequency DBS (5–20 Hz) while in the present investigation subjects received high-frequency DBS (>90 Hz). Thus, local increase in glucose metabolism through DBS occurs likely during both high- and low-frequency stimulation.

Our finding somehow seems to contradict the former notion that DBS exerts a functional lesion in this area [21]. However, there is still no consensus whether high-frequency DBS results in local inhibition or in fact excitation of brain tissue [22]. A commonly acknowledged perspective is that DBS overrides putatively pathological neural activity [23]. Our results point towards local increase in glucose metabolism; still, it remains elusive whether this increase in metabolism results in phasic excitatory impulses or rather in a tonic activation of neurons that leads to an overriding of neural activity of this area. Apart from inhibition and excitation, recent electrophysiological approaches have suggested that DBS may disrupt abnormal information within stimulation sites [24]. It is conceivable that ongoing interference of cellular firing rate (either resulting in inhibition, excitation or disruption of incoming pathological signals) requires increased glucose metabolism. This in turn implies that DBS does not result in silent neural tissue but rather in a consuming state of activity. Yet, there are numerous unknown cellular and molecular mechanisms of DBS (including effects on glia cells and neurotransmitters) that remain to be explored.

Looking at the whole brain changes between ongoing and switched-off DBS resulted in a heterogeneous effect. While in one subject (subject 3), we observed only a slight increase in metabolism in the orbitofrontal and temporal cortex, the other two subjects (1 and 2) showed a partly contradicting outcome. Subject 1 revealed a wide-spread increase in glucose metabolism involving the parietal cortex as well as the medial, lateral, and orbitofrontal prefrontal cortexes with stimulation ON. Contrary to this, subject 2 showed a pronounced decrease in parietal and occipital cortical brain metabolism while FDG uptake in the thalamus was clearly enhanced with stimulation ON vs. stimulation OFF. Thus, we did not observe a clear common network effect, although increase in prefrontal activity in DBS ON condition was observed in subject 1 and partly subject 3 (but not in subject 2).

The heterogeneity of the global results makes the interpretation of these findings difficult. Considering the existing literature, one possible confounder could be treatment response and thus relative decrease in OCD symptoms. Subject 1 was the only patient that clearly responded to the intervention. In fact, this subject showed an early clinical improvement that withstood a relatively low stimulation amplitude of 3.3 Volts (see [10]). In this context, other studies could show that treatment response for OCD was accompanied by an increase in prefrontal glucose metabolism [25]. Congruent to this, response to DBS of the VC/VS correlated with increased prefronto-striatal functional connectivity [6] and increase in prefrontal theta oscillations [26]. We argue that in our subjects, response to the intervention may contribute to the heterogeneity of the results, with the only full responder displaying increased prefrontal activity ratio in ON vs. OFF condition. Notably, PET Scans in the DBS OFF condition revealed a relative prefrontal hypometabolism compared to age-matched healthy participants. Thus, successful DBS may in fact reduce this pathologic prefrontal hypometabolism.

Another possible explanation for the differential changes in glucose metabolism may result from the location of leads and the corresponding VTAs. In fact, differential changes in metabolism dependent on the employed DBS contacts have been described before [7]. In subject 3 we did not observe a clear change in brain metabolism between ON and OFF condition. As shown in Figure 1, VTAs in this subject were located most ventrally, whereas VTAs of subject 2 were located most caudally. The ventral capsule carries fibres to the entire prefrontal cortex through a complex and individual structural organization [27]. Hence, spatial differences of VTAs may also partly explain differences across subjects. Adjusting parameters in subject 2 and 3 may have resulted in better clinical outcome and thus similar changes in metabolism, although this data was not obtained. Overall, our results fit in the heterogeneous and contradicting reports on DBS induced changes in global brain metabolism [7,9] and underlie the possible influence of clinical improvement and localization of stimulation sites.

Another conceivable assumption can be derived from OFF condition measurements of glucose metabolism and clinical outcome. As presented in Figure 2, subject 1 revealed the most evident prefrontal hypometabolism compared to healthy subjects. Within the three subjects, this patient also showed the strongest improvement of OCD symptoms during DBS. Although it cannot be concluded from our data, one might speculate if specific preoperative metabolism patterns might indeed be useful to predict outcome of this invasive and costly procedure. The potential of FDG-PET as a tool to predict response to pharmacotherapy and psychotherapy for OCD has been shown before [25]. Considering that up to now there are no reliable preoperative treatment predictors [28], this observation might be relevant for further research. Of note, this interpretation is based on the assumption, that the OFF condition metabolism can be linked to patients’ baseline brain metabolism. However, in this study, we did not assess preoperative FDG-PET as an actual baseline measurement. It has also been shown before that the implantation of DBS electrodes leads to changes in brain metabolism [29] independently of ongoing electric stimulation. These effects have mostly been linked to an insertional microlesion effect. Thus, our OFF condition results may be influenced by such a microlesional effect. Clinically, the insertion (or microlesion) effect is commonly observed in patients with PD, with an initial transient postoperative improvement of symptoms that usually declines within weeks [30]. The aforementioned animal study [29] assessed FDG PET within two weeks after surgery. In contrast, another study in humans did not observe differences between baseline and DBS OFF glucose metabolism [9]. Noteworthy, this sample underwent FDG PET 28 to 123 days after surgery. This indicates that the influence of the insertion effect on glucose metabolism (like the clinical effects) vanishes over time. In our sample, we assessed FDG PET at least six months after surgery. Although it cannot be assured in our study due to the lack of baseline measurements, we argue that the insertion effect on brain metabolism, if present, would be less pronounced in our sample. Nonetheless, the putative usefulness of preoperative prefrontal hypometabolism as a predictor for treatment response requires specific prospective investigations including baseline measurements. In this context, one has to keep and mind that our study compares brain metabolism with and without *ongoing* DBS and not with and without DBS. Hence, the DBS OFF condition does not constitute a true baseline condition.

The major and most overt limitation of this study is the very small sample size and the consequently missing statistical group analysis. The small sample size resulted mostly from low acceptance rates of the experimental protocol within the patient sample as both exposition to radiation and the risk of relapse of symptoms during the OFF condition discouraged patients to participate to the study. Another limitation is the lack of a healthy control sample. To partly overcome this limitation, we employed publicly available healthy cohorts to compare brain activity that are used in real-life clinical FDG-PET investigations. Further limitations relate to the modelling of VTAs. Although the employed model reflects one of the state-of-the-art approaches, it is inherently an approximation of the actual anatomical expansion of the electric fields, as several influencing factors (e.g., local fiber orientation, subject-specific neural alterations) are not taken into account. In the study design, neither patients nor investigators where blinded with regard to ON/OFF conditions. Thus, a placebo/nocebo effect cannot be ruled out. Differences in medication status may have also influenced brain metabolism, however differences between OFF and ON conditions should not be influenced by this and medication remained stable for months.

## 5. Conclusions

Notwithstanding the small sample size, our study revealed some important insights into how DBS for OCD changes brain metabolism both locally and globally. Using state-of-art methodology to locate electrodes and model individually applied electric fields, we were able to specifically test how glucose brain metabolism is directly affected with high-frequency DBS at stimulation sites. Specifically, our results revealed heterogeneous global changes of brain metabolism including DBS-induced prefrontal increase in glucose uptake, possibly influenced by the degree of improvement of OCD symptoms as well as localization of VTAs. Locally, we observed that high-frequency DBS induced a significant increase in brain metabolism within the individually applied electric fields.

## Figures and Tables

**Table 1:** Demographic data and stimulation settings and current psychotropic medication.

Subject	Sex	Age at Surgery	Preoperative Y-BOCS	Postoperative Y-BOCS	Stimulation Settings	Medication
1	Male	47	28	15	3−, 2−, c+; 11−, 10−, C+; 130 Hz; 3.3V; 120µs	Clomipramine 225mg/d Quetiapine 400mg/d
2	Male	45	37	31	2−,1−, c+;10−,9−, c+; 130 Hz; 4.8V; 150µs	Venlafaxine 225mg/d Mirtazapine 30mg/d
3	Female	54	34	33	3−, 2−, c+; 11−, 10−, C+; 130 Hz; 4.2V; 90µs	Fluoxetine 80mg/d

## Supplementary Materials

The following are available online at https://www.mdpi.com/2076-3425/9/9/220/s1, Figure S1: Close view of individual electrode localization, Figure S2: Glucose hypermetabolism during stimulation OFF condition compared to an age-matched healthy control cohort
