# Supplementary material for: Local and Global Changes in Brain Metabolism during Deep Brain Stimulation for Obsessive-Compulsive Disorder
Source: Brain Sci. 2019 Aug 30;9(9):220. doi: 10.3390/brainsci9090220 (PMC6770477; doi:10.3390/brainsci9090220)
Supplement: Supplementary file 1 [file brainsci-09-00220-s001.pdf]

brain sciences

MDPI

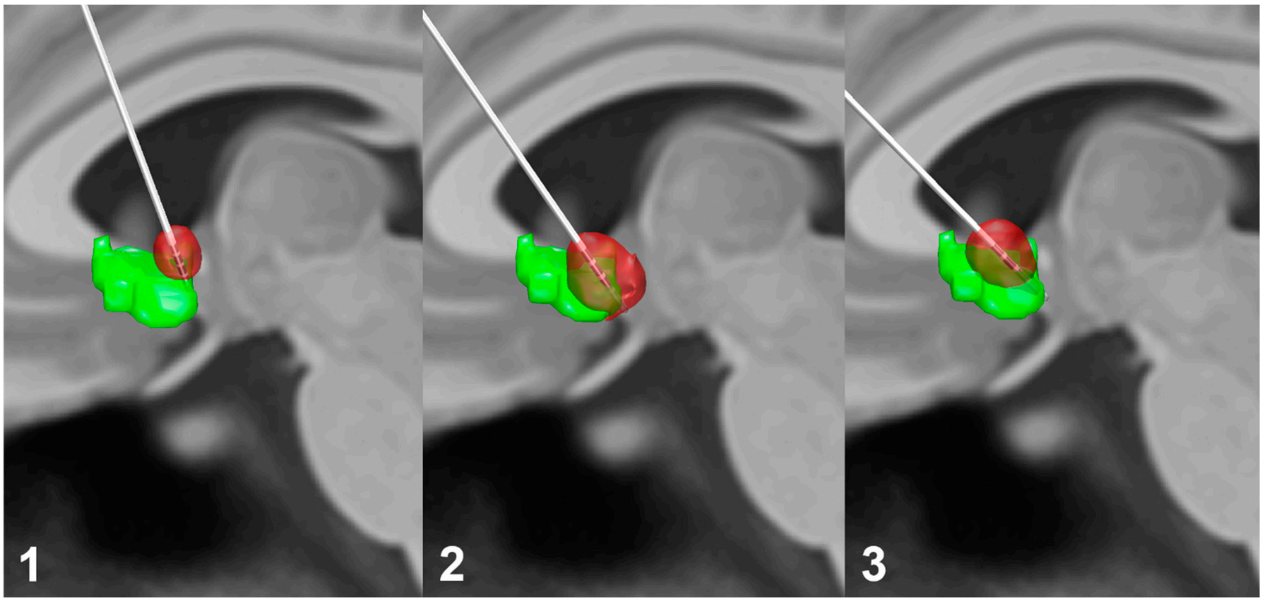

Figure S1: ***Close view of individual electrode localization*** *with corresponding volumes of activated tissue (red) at time of imaging acquisition. More distal contacts were implanted in the ventral striatum with the nucleus accumbens (green), more proximal contracts were located in the ventral capsule. For display purposes, only the left hemisphere is shown.*

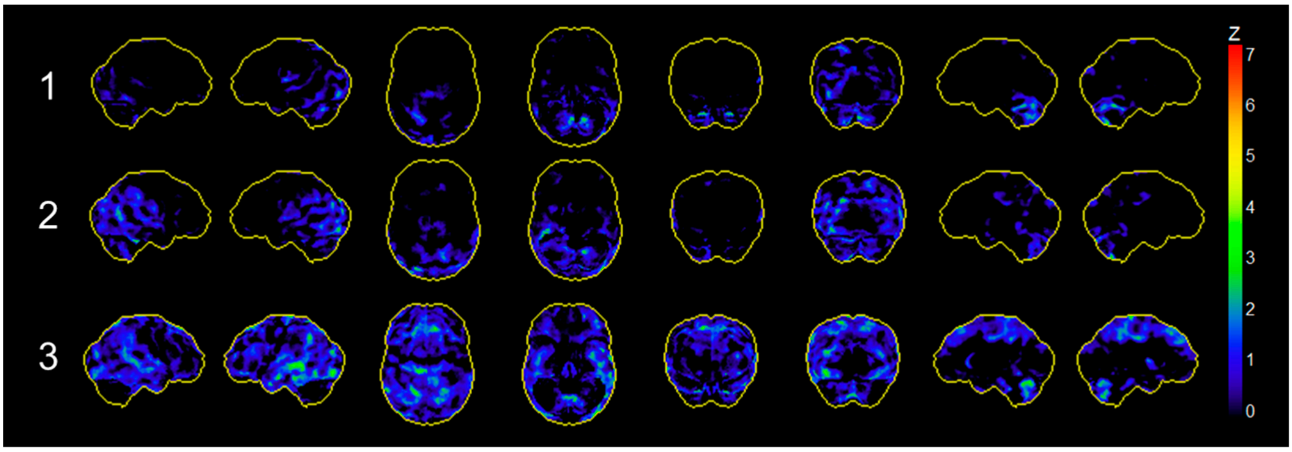

Figure S2: ***Glucose hypermetabolism during stimulation OFF condition compared to an age-matched healthy control cohort.*** *Overall, there was no distinct global hypermetabolism across subjects (z > 2). Only subject three showed a slight hyperactivity in the right temporal cortex and cerebellum.*
